# Biochemical Examination of Plasma Ghrelin Levels in Individuals Afflicted With Chronic Periodontal Disease: A Comparative Study

**DOI:** 10.7759/cureus.56536

**Published:** 2024-03-20

**Authors:** Neelam Das, Pavan Kumar Addanki

**Affiliations:** 1 Department of Periodontology, Rama Dental College Hospital & Research Centre, Kanpur, IND; 2 Department of Periodontology, Kamineni Institute of Dental Sciences, Narketpally, IND

**Keywords:** alkaline phosphatase (alp), osteocalcin, ghrelin, pro-inflammatory cytokines, chronic generalized periodontitis

## Abstract

Objective: This study intended to assess plasma ghrelin levels in individuals with chronic periodontitis and analyze potential associations with bone turnover indicators, serum cytokines, and periodontal parameters.

Material and methods: The research contained 80 patients each with 40 individuals with periodontally healthy controls (C) (28 males, 12 females) and 40 chronic periodontitis (CP) patients (29 males, 11 females). The blood samples were analyzed for soluble receptor activator nuclear factor kappa B ligand (sRANKL), interleukin-1 beta (IL-1β), total and acylated ghrelin, tumor necrosis factor-alpha (TNF-α), osteocalcin (OSC) and alkaline phosphatase (ALP), and periodontal parameters were recorded.

Results: The CP group had considerably higher plasma concentrations of both acylated and total ghrelin than the C group (p<0.05). Gender-based investigation showed substantial differences only among men in both groups (p<0.05). Hence, no significant modifications were identified in serum sRANKL, TNFα, and ALP levels between the groups. However, there was a notable difference in serum OSC and IL-1β levels in the CP group (p<0.05). Furthermore, total ghrelin/acylated ghrelin and total ghrelin/ALP revealed positive correlations. No significant association was found between symptoms and ghrelin levels.

Conclusion: The study findings indicate elevated levels of ghrelin and acylated ghrelin in male CP patients.

## Introduction

Ghrelin, a peptide hormone that is mostly secreted from the stomach but present in various tissues, cells, and organs, exists in two main types: acylated and des-acylated. Its physiological roles encompass food intake, growth hormone production, and energy metabolism [1].

Beyond its established functions, ghrelin has been identified as a regulator of the immune system and bone metabolism. It has anti-inflammatory properties by inhibiting the generation of pro-inflammatory cytokines, promoting bone formation, and fostering osteoblast differentiation [2]. Ghrelin levels are influenced by various health conditions; inflammatory diseases like ankylosing spondylitis and inflammatory bowel disease elevate ghrelin levels, but Crohn's disease, metabolic syndrome, obesity, and type 2 diabetes, decrease them. Periodontitis, a chronic illness leading to tooth loss, can impact ghrelin levels due to its systemic effects on the body [3].

The current investigation aims to estimate ghrelin levels in the bloodstream among individuals with chronic periodontitis (CP). Additionally, it sought to identify potential correlations between ghrelin levels and other clinical periodontal indicators, including serum bone-turnover markers and serum cytokine levels.

## Materials and methods

Study population

This was a cross-sectional study conducted between July 2023 and Dec 2023. This research involved 80 participants aged between 29 and 42 years, comprising 40 individuals with periodontally healthy controls (C) (28 males, 12 females) and 40 CP patients (29 males, 11 females). The subjects were selected from individuals looking for dental care at the Periodontology Department, Rama Dental College Hospital & Research Centre, Kanpur, India.

Exclusion criteria encompassed individuals with current or past smoking habits, being pregnant, who are lactation, in menopause, antibiotic use (within the past three months), hormonal replacement therapy, anti-inflammatory drug usage or immunomodulation, recent periodontal therapy (within the past six months), major systemic diseases, or aggressive periodontitis.

Participants underwent a thorough systemic examination, including assessments of serum lipid profiles and blood glucose levels. Fasting blood glucose (FBG) levels and serum lipid profiles were analyzed in the institutional laboratory. Relevant data such as body mass index (BMI) (kg/m^2^), gender, and age were also reported.

Inclusion criteria were set as follows: FBG below 110 mg/dL; BMI between 18.5 and 25; triglycerides (TRG) below 200 mg/dL and total cholesterol (TC) below 200 mg/dL; low-density lipoprotein cholesterol (LDL) below 130 mg/dL; high-density lipoprotein cholesterol (HDL) above 35 mg/dL.

The study protocol was approved by the Institutional Review Board (IRB) of Rama Dental College Hospital & Research Centre (RDCHRC/ETHICSCOMMITTE/0157). Participants were given comprehensive information about the study before giving their written consent.

Periodontal parameters

The periodontal examination included the recording of various clinical parameters by the same clinician Williams periodontal probe (Hu-Friedy, Chicago, IL, USA) was used. The recorded parameters were as follows: (i) plaque index (PI) assessed according to the method outlined in the study by Silness and Loe [4]; (ii) gingival index (GI)* *evaluated based on the criteria specified in the study by Loe and Silness [5]; (iii) probing depth (PD) recorded using the periodontal probe to measure the depth of periodontal pockets; (iv) clinical attachment levels (CALs) assessed by an oral surgeon to ascertain each tooth's distinctive level of attachment loss; (v) bleeding on probing site percentage (BOP %) measured to determine the proportion of sites exhibiting BOP.

 Subjects with recorded periodontal parameters were categorized into two groups: those diagnosed with CP and those exhibiting periodontal health (C). A diagnosis has been determined using the radiographic and clinical criteria stated in the Classification of Periodontal Diseases Consensus 1999 [6]. For individuals with CP, the criteria included having at least 14 teeth, with over 30% of sites displaying a “probing pocket depth (PPD)≥4 mm and CAL ≥4 mm.” Individuals who had excellent periodontal health had a mean BOP≤ 25%, a mean GI<1, and no attachment loss sites.

Laboratory analysis

After an overnight stay in bed, venous blood was obtained between 9:00 and 10:00 am the following morning, before breakfast. The blood samples were subjected to tests for various biochemical markers, including TNF-α, sRANKL, IL-1β, osteocalcin (OSC), alkaline phosphatase (ALP), acylated ghrelin, and total ghrelin.

In order to determine the amounts of acylated and total ghrelin, two milliliters of venous blood was collected, and aprotinin was added. After centrifugation, supernatants were collected, and aprotinin and hydrochloric acid were added. Commercial ELISA kits (Total ELISA kit/Millipore Human Ghrelin Active, USA) were used to determine plasma levels.

Serum levels of TNF-α, sRANKL, OSC, and ALP were assessed using 8 mL of venous blood. After centrifugation, specimens were stored and measured with commercial ELISA kits. ALP and OSC levels were determined using a spectrophotometer and electrochemiluminescence immunoassay methods, respectively.

Statistical analysis

NCSS/PASS 2000 Dawson Edition statistical software (NCSS, Kaysville, UT) was employed to perform power analysis. The study aimed for a statistical power exceeding 70%, a standardized difference of 0.50 and a significance level of 0.05.

IBM SPSS Statistics for Windows, Version 15 (Released 2006; IBM Corp., Armonk, New York, United States) was employed to perform the statistical analysis. Normality distribution and equality variances were calculated. An independent t-test was employed for group comparisons. A two-level factorial model was employed to estimate the influence of both gender and periodontal health on ghrelin levels. Risk estimation and Chi-square tests were utilized to evaluate variables, and Pearson’s correlation analysis was applied for correlation assessments. The quantitative data were shown as mean ± standard deviation, and the statistical significance level was determined at p<0.05.

## Results

This study has 80 participants, including 40 individuals with periodontally healthy controls (C) (28 males, 12 females) and 40 CP patients (29 males, 11 females). All participants completed the assessments. In the CP and C groups, the mean ages were 38.96 ± 3.88 and 36.74 ± 4.82 years. Table 1 displays the parameters of the research groups, showing no substantial differences in BMI, gender, age, LDL, HDL, TRG, FBG, and TC across the groups (p > 0.05).

**Table 1:** Characteristics of study participants (mean ± SD) by Chi-square tests. C: Systemically and periodontally healthy control group, CP: chronic periodontitis group, BMI: body mass index, FBG: fasting blood glucose, TC: total cholesterol, TRG: triglyceride, HDL: high-density lipoprotein, LDL: low-density lipoprotein

	C (n = 40) (12F/28 M)	CP (n = 40) (11F/29 M)	p-value
Age ( years)	36.74 ± 4.82	38.96 ± 3.88	0.098
BMI (kg/m^2^ )	27.95 ± 2.21	23.30 ± 1.90	0.330
FBG (mg/dL)	94.47 ± 6.93	98.74 ± 7.96	0.077
TC (mg/dL)	169.90 ± 29.48	169.17 ± 27.32	0.630
TRG (mg/dL)	95.91 ± 39.45	98.20 ± 37.30	0.986
HDL (mg/dL)	50.97 ± 9.38	50.97 ± 12.64	0.878
LDL (mg/dL)	99.57 ± 35.54	100.19 ± 44.88	0.733

The periodontal features of the research groups are displayed in Table 2, where it is evident that the C group had considerably decreased levels of all clinical markers than the CP group (p<0.05). Men showed considerably higher CAL and PD than women in the CP group (p<0.05). Hence, gender differences did not appear in the C group (p>0.05).

**Table 2:** Independent t-test was employed for periodontal clinical parameters in study groups (mean ± SD). C: Systemically and periodontally healthy control group, CP: chronic periodontitis group, PI: plaque index, GI: gingival index; BOP%: bleeding on probing, PD: probing depth, CAL: clinical attachment level. Statistically significant difference was shown in bold (p˂0.05)

Periodontal parameters	C (n = 40) (12F/28 M)	CP (n = 40) (11F/29 M)	p-value
GI (mm)	0.16 ± 0.09	1.31 ± 0.37	0.000
PI (mm)	0.25 ± 0.08	2.13 ± 0.66	0.000
BOP %	6.97 ± 5.75	93.73 ± 11.02	0.000
PD (mm)	1.68 ± 0.17	4.15 ± 0.87	0.000
CAL (mm)	1.66 ± 0.15	4.91 ± 0.44	0.000

Serum data for the research groups and gender-specific subgroups are shown in Table 3, wherein the CP groups exhibited lower serum OSC and greater levels of IL-1β in comparison to the C groups (p<0.05). Male categories were the main ones where significant differences of p<0.05 were found. Consequently, there was no discernible change (p>0.05) in the levels of ALP, TNF-α, and sRANKL between any of the groups or subgroups.

**Table 3:** Independent t-test was employed for serum parameters in study groups and subgroups stratified by gender (mean ± SD). C: Systemically and periodontally healthy control group, CP: chronic periodontitis group, ALP: alkaline phosphatase, OSC: osteocalcin, sRANKL: soluble receptor activator of nuclear factor-kappaB, IL-1β: interleukine-1β, TNF-α: tumor necrosis factor-α. Statistically significant differences are shown in bold (p˂0.05)

Serum parameters	Gender	n (C/CP)	C	CP	p-value
ALP (U/L)	Total	40	59.14 ± 16.63	60.91 ± 15.71	0.641
	Female	12/11	51.55 ± 12.02	57.00 ± 15.44	0.416
	Male	28/29	63.26 ± 18.41	63.00 ± 15.62	0.954
OSC (ng/mL)	Total	40	18.00 ± 5.49	14.68 ± 3.49	0.004
	Female	12/11	16.84 ± 5.26	14.92 ± 1.46	0.168
	Male	28/29	18.64 ± 5.06	15.03 ± 4.10	0.010
sRANKL(pg/mL)	Total	40	194.49 ± 114.01	235.17 ± 125.81	0.165
	Female	12/11	215.87 ± 101.98	206.36 ± 79.66	0.789
	Male	28/29	185.91 ± 119.39	248.92 ± 149.60	0.111
IL-1β (pg/mL)	Total	40	78.59 ± 85.09	163.13 ± 190.01	0.016
	Female	12/11	84.37 ± 64.25	111.00 ± 105.36	0.437
	Male	28/29	76.46 ± 86.31	189.47 ± 217.63	0.025
TNF-α (pg/mL)	Total	40	1.11 ± 0.78	1.55 ± 1.51	0.165
	Female	12/11	1.18 ± 0.86	0.99 ± 0.88	0.551
	Male	28/29	1.07 ± 0.73	1.81 ± 1.56	0.072

As shown in Table 4, significant differences were mostly noted between the male categories (p<0.05), with acylated and total ghrelin levels higher (p<0.05) in the CP group than in the C group. The female patients in the CP group had greater and lesser levels of acylated and total ghrelin, respectively, than the female patients in the C group, although the differences were not statistically significant (p>0.05). In contrast, the CP group's male participants exhibited elevated levels of both acylated and total ghrelin.

**Table 4:** Independent t-test was employed for levels of total and acylated ghrelin in study groups and gender subgroups (mean ± SD). C: systemically and periodontally healthy control group, CP: chronic periodontitis group Statistically significant differences are shown in bold (p˂0.05)

Serum parameters	Gender	n (C/CP)	C	CP	p-value
Total ghrelin (pg/mL)	Total	40	392.27 ± 213.30	515.61 ± 298.96	0.050
	Female	12/11	481.07 ± 203.54	501.46 ± 340.48	0.962
	Male	28/29	342.93 ± 203.69	521.18 ± 282.62	0.019
Acylated ghrelin (pg/mL)	Total	40	159.12 ± 71.36	199.54 ± 74.25	0.047
	Female	12/11	187.69 ± 66.14	164.60 ± 55.21	0.497
	Male	28/29	142.69 ± 76.05	212.19 ± 74.95	0.003

An analysis of variance using a two-level factorial model was employed to examine the impact of gender on ghrelin levels, Table 5 reveals the statistically significant differences for acylated ghrelin levels (p<0.05) rather than for total ghrelin levels (p>0.05), indicating an association between gender and periodontal conditions.

**Table 5:** Participant characteristics from two-level factorial analysis of the variance model (mean ± SD) by the independent t-test. C: systemically and periodontally healthy control group, CP: chronic periodontitis group Statistically significant differences are shown in bold (p˂0.05)

Dependent variable	Gender	Group	n	Mean	Standard deviation	%95 Confidence Interval		p-value
						Lower bound	Upper bound	
Total ghrelin (pg/mL)	Female	-	23	493.27	259.40	385.70	599.88	0.373
	Male	-	57	442.05	268.18	376.86	506.25	
	-	C	40	414.00	274.90	323.23	507.27	0.141
	-	CP	40	514.32	279.06	418.49	609.17	
	Female	C	12	482.07	259.14	336.29	633.86	0.243
		CP	11	505.46	259.13	348.06	667.86	
	Male	C	28	353.92	268.16	256.46	461.39	0.241
		CP	29	523.18	259.13	417.98	629.39	
Acylated ghrelin (pg/mL)	Female	-	23	177.64	77.59	149.25	207.03	0.967
	Male	-	57	187.44	80.54	166.89	199.98	
	-	C	40	166.19	75.31	141.12	194.26	0.194
	-	CP	40	198.89	85.96	173.26	254.53	
	Female	C	12	188.69	77.53	149.04	239.34	0.013
		CP	11	195.60	80.51	133.14	228.06	
	Male	C	28	147.79	71.75	115.53	179.17	0.013
		CP	29	242.19	80.55	193.74	266.97	

Two groups of participants were subsequently formed based on median levels of higher or lower total/acylated ghrelin for additional analysis. Risk estimation and Chi-square tests demonstrated a link between elevated levels of acylated and total ghrelin and periodontitis (Table 6).

**Table 6:** Results of chi-square tests and risk estimation for total and acylated ghrelin levels. * Chi-square test C: Systemically and periodontally healthy control group, CP: chronic periodontitis group Statistically significant differences are shown in bold (p˂0.05)

Independent variable	Total ghrelin (pg/mL)		p-value*	Odds ratio (OR) (%95 CI)	Acylated ghrelin (pg/mL)		p-value*	Odds ratio (OR) (%95 CI)
		˂367.4			≥367.4					˂166.9	≥166.9		
Periodontal condition								
C	20 (57.1)	15 (42.9)	0.232	1	21 (60.0)	14 (40.0)	0.094	1
CP	15 (42.9)	20 (57.1)		1.98 (0.91–6.79)	14 (40.0)	21 (60.0)		3.45 (0.97–6.76)

Total ghrelin and ALP showed an association that was significant in the group with CP (p = 0.038, r = 0.352). Furthermore, acylated and total ghrelin revealed a significant correlation (p = 0.041, r = 0.347). No correlations were found to be significant (p>0.05) identified between inflammatory indicators in serum or periodontal clinical parameters and total and acylated ghrelin levels.

## Discussion

In this research, we investigated the plasma concentrations of acylated and total ghrelin in individuals with CP and explored their connection with clinical periodontal measures, serum indicators of inflammation and bone turnover, and levels of both total and plasma acylated ghrelin.

Pro-inflammatory cytokine production and inflammatory cell proliferation have been shown to be inhibited by ghrelin. It stimulates IL-10 while suppressing neutrophil accumulation and IL-1β, TNF-α, and IL-8 production. Ghrelin also improves endothelial dysfunction in rat endothelial cells and has anti-inflammatory effects in human endothelial cells and smooth muscle against LPS. Ghrelin treatment is reported by Kodama et al. to decrease myeloperoxidase and neutrophil accumulation and TNF-α and IL-8 production [7]. Exogenous ghrelin administration has also been shown to increase IL-10 production while suppressing TNF-α and IL-1β production. Furthermore, it was shown that ghrelin activation of T-cells increased the amounts of IL-13 and IL-4 proteins [8]. Ghrelin treatment in rat endothelial cells reduces endothelial dysfunction, as demonstrated by Shimizu et al. [9]. Chow et al. found that ghrelin, upon exposure to LPS, elicits an anti-inflammatory response in human vascular endothelial cells and smooth muscle, suggesting its potential therapeutic role in mitigating inflammation-related cardiovascular diseases [10]. A study discovered that ghrelin is produced by oral fibroblasts and epithelium and that it inhibits oral epithelial cells stimulated by LPS or TNF-α from synthesizing IL-8. Ghrelin is found in higher amounts in the gingival crevicular fluid [11].

With the exception of ghrelin levels measured in salivary, periodontal tissues and GCF among individuals without periodontitis, this is, as far as we are aware, the initial study in India in individuals suffering from CP, to evaluate the amount of circulating acylated and total ghrelin [12]. Our findings showed that, in contrast to the C group, the CP group had greater levels of both total and acylated ghrelin.

According to Hataya et al., ghrelin levels were lowered during the early phase of LPS treatment, but they increased with repeated LPS administration [13]. Furthermore, it has been documented that inflammatory ailments like celiac, ankylosing spondylitis, and inflammatory bowel disease are associated with elevated ghrelin levels. According to Pöykkö et al., there is a positive correlation between plasma ghrelin levels and the early stage of atherosclerosis development [14]. It has been proposed that ghrelin expression is induced by the pro-inflammatory cytokines production during inflammation. TNF-α and IL-6 levels are strongly linked with the serum total ghrelin content, as demonstrated by Mafra et al. [15]. Sung et al. observed that circulating ghrelin levels were impacted by systemic TNF-α modification [16]. On the other hand, some illnesses including obesity, metabolic syndrome, and type 2 diabetes reduced the levels of plasma ghrelin.

Research indicates that levels of circulating ghrelin may be influenced by cytokine production and inflammation, but the severity and disease progression phase affect ghrelin levels. Most studies only evaluated total ghrelin levels, neglecting acylated or des-acylated ghrelin levels for anti-inflammatory activity [17]. Thus, in our investigation, acylated ghrelin levels were taken into account in addition to total ghrelin.

CP significantly influences levels of both acylated and total ghrelin, with higher levels in the CP group. However, these levels do not correlate with clinical periodontal parameters. Previous studies suggest ghrelin's effects are cell-specific, and a study comparing gingival tissue ghrelin levels, plasma ghrelin levels, and/or GCF possibly shed light on the connection between CP and ghrelin levels [18]. Ghrelin is linked to bone metabolism, influencing osteoblast differentiation and proliferation. It also induces OSC and ALP gene expressions and can increase the production of these genes in rat calvarial defects.

Studies have shown no direct link between ghrelin and bone metabolism parameters like amino-terminal pro-peptides of type 1 collagen or OSC [19]. Nonetheless, ghrelin is inversely connected with sRANKL and positively linked with OPG in individuals with chronic renal impairment, according to a recent research study [20]. In contrast, ghrelin and OPG or RANKL did not correlate in postmenopausal women, according to Di Carlo et al. [21].

Total ghrelin and ALP were observed to positively correlate in patients with CP, but not with OSC or sRANKL. The positive correlation in periodontal disease may be correlated with bone metabolism. Further evaluation is needed based on severity and aggressiveness. The study's conclusions on the impact of ghrelin on individuals with chronic periodontitis' sRANKL/OPG ratio were not reached due to the lack of OPG assessment.

Studies show ghrelin levels are influenced by factors like age, gender, and BMI, with conflicting findings on gender effects, with some suggesting no influence and others suggesting higher levels in women [22].

 The acylated and total ghrelin levels of the women in the C group were higher; therefore, the study found no statistically significant differences. Additionally, although not statistically significant, it was shown that the CP group's levels of acylated ghrelin when compared to the C group were lower. The small number of female participants and the males in the CP group having considerably greater levels of acylated and total ghrelin than the men in the C group are among the study's limitations.

Periodontal and gender status significantly influence ghrelin levels, with acylated ghrelin linked to gender in periodontitis cases, suggesting sex hormones and inflammation regulate ghrelin expression differently in women and men. In accordance with our study, Cicero et al. noted, in line with our proposal, that ghrelin interacted specifically with sex hormones and that one of the primary factors influencing ghrelin levels in females was the ratio of testosterone to estrogen [23]. Additionally, ghrelin and testosterone showed a favorable correlation in both postmenopausal women and men, according to Greenman et al. [24].

Age and gender have an impact on plasma ghrelin levels. Aside from a negative connection, there is no evidence of a link between ghrelin and age. On the contrary, ghrelin and age were found to positively correlate, according to Purnell et al. [22]. We observed no association between age and levels of plasma ghrelin in any of the research subjects, who were all between the ages of 29 and 42.

This research sheds light on the association between levels of ghrelin and CP, emphasizing the importance of considering both total and acylated forms. The study highlights the need for further investigation, especially regarding the specific mechanisms by which ghrelin influences periodontal health and the interplay between gender, inflammation, and ghrelin expression.

## Conclusions

The current research reveals elevated ghrelin levels in CP patients, particularly in males, while females exhibit reduced acylated ghrelin levels. The findings propose a potential gender and inflammation activity influence on ghrelin. The study underscores the necessity for additional research to comprehend ghrelin's involvement in the development of periodontitis. Furthermore, a comparative analysis of plasma ghrelin levels with those in saliva, gingival tissue, and gingival crevicular fluid across varying periodontitis severities could offer valuable insights into ghrelin activity.
